# Strong Second Harmonic Generation and Nonlinear Optical Activity in Chiral Supramolecular Polymers

**DOI:** 10.1021/acs.jpclett.5c02495

**Published:** 2025-12-11

**Authors:** Carlos H. D. dos Santos, Marcelo G. Vivas, Filipe A. Couto, Guy Koeckelberghs, Cleber R. Mendonça, Leonardo De Boni

**Affiliations:** † Instituto de Física de São Carlos, 117186 Universidade de São Paulo, São Carlos 13566-590, SP, Brazil; ‡ Laboratório de Espectroscopia Óptica e Fotônica, Universidade Federal de Alfenas, Poços de Caldas 37701-840, MG, Brazil; § Laboratory of Macromolecular and Physical Organic Chemistry, Katholieke Universiteit Leuven, Celestijnenlaan 200F, Box 2404, B-3001 Heverlee, Belgium

## Abstract

The growing demand for advanced optical materials has made nonlinear photonics a key area of research, particularly for developing new technologies. Therefore, this study focuses on chiral supramolecular structures, which are highly promising due to their exceptional second-order optical susceptibility (χ^(2)^) values. In particular, we investigated the origin and optical activity of second harmonic generation (SHG) in chiral polybinaphthalenes using femtosecond laser pulses. Our outcomes reveal a high χ^(2)^ value, up to 7 pm/V, influenced by the attached chromophores at the polymer backbone. Structural analysis showed rodlike/helical structures that promote directional preference for the SHG signal. Simulations unveil that the SHG process is connected to the quasi-phase matching process, and the material’s robust chiral structure leads to high optical activity observed via lock-in amplifier detection. In conclusion, the conjunction of high SHG values and optical activity of these supramolecular structures makes them excellent candidates for chiral photonics applications.

## Graphical abstract

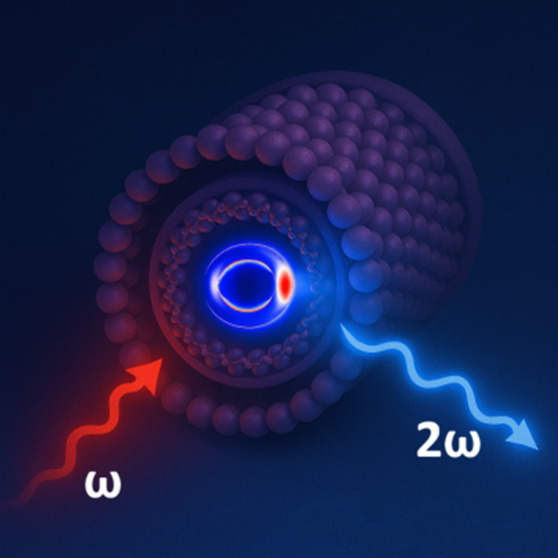

Chiral functionalized polymers have garnered particular interest, due to their straightforward capacity to conceive noncentrosymmetric supramolecular structures with high nonlinear polarizabilities.^1–5^ This performance renders them outstanding candidates for applications involving second-order nonlinear optical (NLO) responses such as frequency conversion,^6^ optical storage devices,^7,8^ and electro-optic devices,^9,10^ and the new field of chiral photonics.^11,12^ Over the past few decades, significant efforts have been directed toward investigating and progressing on enhancing the NLO response at supramolecular systems, and notable evolution has been accomplished in the structure–property relationships, especially in periodically poled polymers (PPP).^13–15^ The introduction of the suitable isolation group^16^ (SIG) concept has been responsible for a pioneering design, e.g., spherical,^17^ H-shaped,^18^ star-type,^19^ and hyperbranched,^20^ that enhances the signal of second harmonic generation (SHG). On the other hand, synthesizing these supramolecular geometries requires complex and high-cost chemistry. The fabrication of PPP involves meticulous steps, including elevated temperatures and an intensive external electrical field. Besides, NLO chromophores attached to backbone polymers generally have a D-π-A (donor/acceptor) structure and typically exhibit a rodlike structure, leading to robust intermolecular dipole–dipole interactions within the supramolecular system. This characteristic significantly complicates the alignment of chromophores into a noncentrosymmetric arrangement induced by the poling process.^21,22^ In addition, experimental measurements in colloidal systems using the SHG technique^23^ allow us to determine the magnitude of the frequency upconversion in organic molecules,^24–27^ as well as nanocrystal and bulk semiconductors (NCs).^28–31^ Few studies reported SHG in supramolecular systems.^32^ In this context, recognizing the significance of performing the nonlinear optical properties of the supramolecular materials, herein, we conducted a novel study of frequency-resolved second-order nonlinearities in novel samples of chiral donor-embedded polybinaphthalenes (CDEP) in dimethyl sulfoxide (DMSO) solution. In general, this class of polymer has a noncentrosymmetric twisted and rigid structure, such as a rodlike helical structure. The chirality and the chromophores are introduced to promote the increase in the nonlinear optical response and add new optical phenomena, as demonstrated by critical results in the literature.^33,34^ In particular, chirality plays a fundamental role in SHG imaging^35–38^ due to the selectivity of the circularly polarized pump light^39^ and is crucial to providing information about the morphology and three-dimensional orientation of the molecules, including sensors and quantum optics applications.^39–42^

With this in mind, we present a SHG and optical activity frequency-resolved experimental study using a tunable femtosecond amplified pulsed laser. Atomic force microscopy (AFM) and dynamic light scattering (DLS) were also employed to untangle structural information on polymers in solid-state and solution, respectively, such as the size and shape of the CDEP. AFM images unveil that the polymers present a hollow cylinder structure with a diameter of hundreds of nanometers, which agrees with the measures of DLS in the solution. Polarization studies performed in solution reveal the macroscopic contribution of the collected signal. Our results suggest that the fundamental origin of SHG may be attributed to quasi-phase matching (QPM) provided by finite-difference time-domain (FDTD) simulations to the helical supramolecular self-assembly, such as PPP systems, enhanced by quantum and classical mechanisms. Finally, the chirality is unveiled through optical activity SHG measurements, such as the difference between left and right polarization intensities, can reach up to 9%.

Figure 1 displays the molecular structure of the chiral supramolecular polymers, based on a triphenylamine group as a core (blue) linked to the two-chiral binaphthalene (S enantiomer) units in gray and functionalized chromophores, represented by red color, as can be seen in Figure 1. The details of the synthesis and linear circular dichroism measurements are described in ref 43. The main difference between the polymers is the functionalized chromophore with distinct electron-withdrawing groups (EWGs) responsible for triggering nonlinearities.

**Figure 1:**
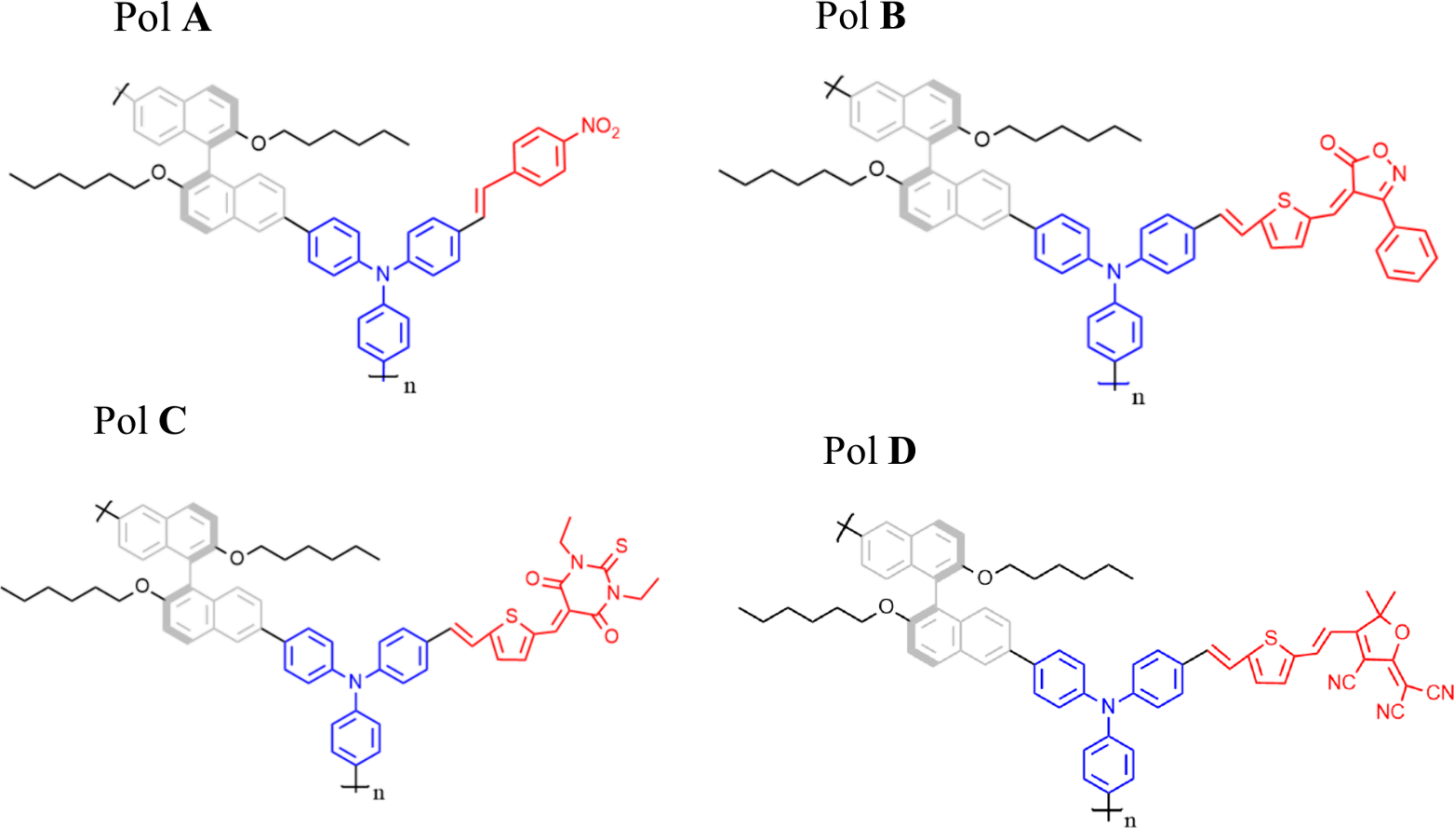
Supramolecular structure of chiral chromophore-functionalized polybinaphthalenes.

It is worth mentioning that χ^(2)^ ∝ *N*μβ*E*, in which *N* is the concentration of chromophore, μ the dipole moment, β the first-order hyperpolarizability coefficient, and *E* the applied electric field. In this case, it implies that the χ^(2)^ signal can be increased using chromophores with higher hyperpolarizabilities and dipole moments or increasing the loading densities. Besides, the attachment of the chromophores as a side chain results in a treelike structure that prevents the undesired centrosymmetrical ordering of the chromophores, but becomes flexible enough to induce the noncentrosymmetry by electrical poling.^44^ The absorption spectra presented in show two main one-photon absorption (1PA) bands at the UV-Vis region for all four polymers dissolved in DMSO solvent. The higher energy absorption band is found at all polymers centered around 350 nm, which is related to the binaphthalene core^44^ and the lowest energy one (LE) to the triphenylamine core attached at chromophores A, B, C, and D centered, respectively, at 433, 545, 561, and 598 nm. No fluorescence at all was observed.

The microscopic dispersion of SHG observed in the Pol A, Pol B, Pol C, and Pol D in DMSO solution is depicted in Figure 2. In , the quadratic dependence of the SHG with the laser intensity is displayed as an example for Pol A at an excitation wavelength of 1200 nm for six different concentrations. Also, in the (correct is ), the SH signal is compared to a small standard molecule (*para*-nitroaniline) in order to quantify its value. Additionally, a previous analysis was performed to confirm whether the double-frequency signal detection has a microscopic or macroscopic derivation. In the SHG experimental setup, a broadband polarizer was placed before the photomultiplier (PMT) for s- and p-polarized detection. The result shows the same signal for both s- and p-polarization, which indicates a macroscopic origin (χ^2^).

**Figure 2:**
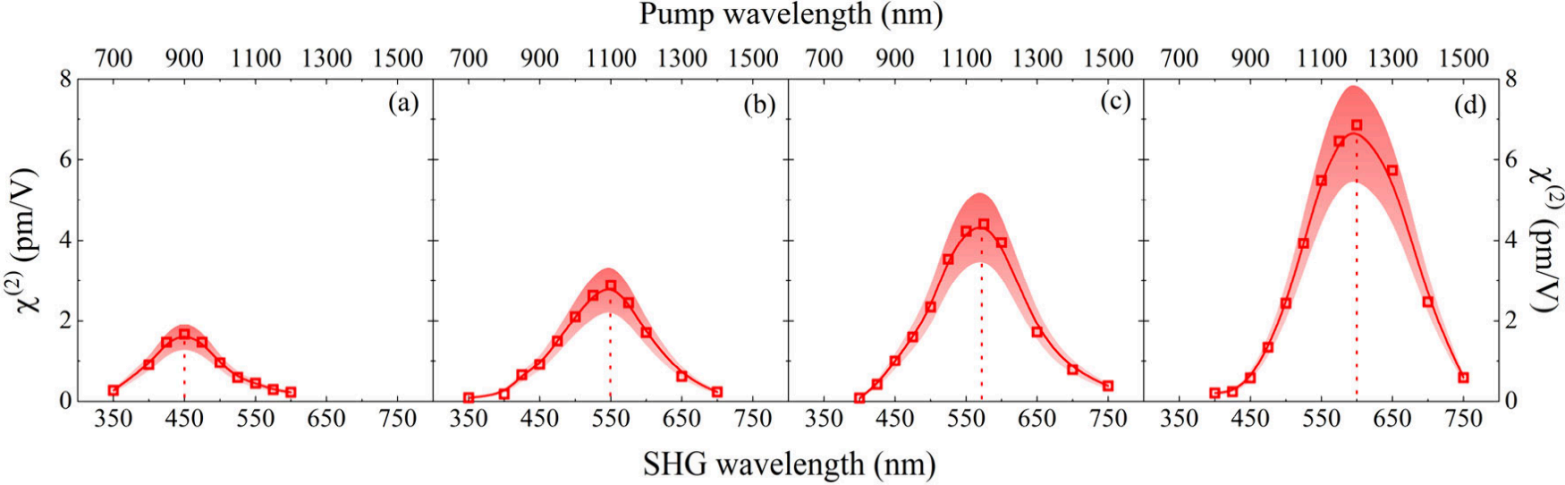
Symbols represent the dispersion of the second-harmonic generation (χ^(2)^) in pm/V units for the CDEP polymers in DMSO solution obtained by the SHG femtosecond technique: (a) A, (b) B, (c) C and (d) D. The solid line is present only to guide the eye.

The χ^2^ dispersion behavior is similar for all samples, in which the values tend to decrease for shorter and longer wavelengths concerning the peak (Pol A = 1.5 ± 0.4 pm/V at 900 nm, Pol B = 2.5 ± 0.7 pm/V at 1100 nm, Pol C = 4.5 ± 1.3 pm/V at 1150 nm, and Pol D = 7.1 ± 2.2 pm/V at 1200 nm). Initially, the high χ^2^ peak values could be attributed to the two-photon absorption (2PA) transition in the near-infrared region,^44^ which promotes enhancement in the SHG signal due to the two-photon resonance, which is described by the second-order time-dependent perturbation theory.^26^ However, these polymers present a moderate 2PA cross-section, compared to other π-conjugated polymers. At the same time, the variance in the SHG maxima, reaching up to 5-fold when comparing Pol A with Pol D, cannot be explained only by considering the 2PA. Another effect would be the different spectral positions of 2PA bands among the polymers,^45,46^ which could favor a resonance enhancement effect on the SHG signal. To understand this, a two-level model (2LM)^47^ simulation for the LE absorption band (chromophore absorption band) was employed to calculate the 2PA enhancement factor that relates to the enhancement of SHG when the frequency of the scattered photon (2ω) is close to the 2PA allowed transition.^48^ Using the second-order perturbation theory, we can clearly see this effect because the denominator in the dynamical term for 2LM is proportional to (ω_01_ – 2ω), in which ω_01_ is the two-photon allowed transition frequency of chromophore (see the ). As shown in (correct is ), similar results for the enhancement factor were found among the polymers, which suggests that the structural effect of polymers plays a fundamental role in the strong SHG signal observed. The curves of the 2PA enhancement factor are depicted in and are calculated by using . In this context, one alternative explanation for the χ^2^ variances was to consider the polymer’s size and shape combined with the 2PA effect. First, dynamic light scattering (DLS) and atomic force microscopy (AFM) measurements were performed to provide the geometrical characteristics of polymers in solution and solid state, respectively. A brief description of both procedures can be found in . Figure 3 (left) shows the AFM topography images of the supramolecular structure of the CDEP polymers on the silicon substrate. The AFM images reveal a hollow cylindrical structure across all polymers, with similar cylinder wall thicknesses of 170 ± 40 nm, average heights of 32 ± 9 nm, and diameters ranging from 400 nm to 800 nm. In Figure 3 (right), we depict the size distribution (cylinder diameter) obtained from the DLS technique. First of all, a good agreement was found when we compared the cylinder diameter obtained from the AFM (solid-state) and DLS (in solution). Such an interesting outcome indicates that the polymers present a geometry similar in the solid-state and solution. As can be seen in DLS measurements, the average size of the size distribution presents a progressive increase, going from 478 nm (Pol A) to 825 nm (Pol D), with an average polydispersity index (PDI) value around 0.3, indicating a considerable level of polydispersity. In general, if PDI > 0.5, the distribution is rather broad and could significantly increase in the linear scattering in solution, which could affect the SHG conversion and consequently the optical activity signal in studied samples.^49^.

**Figure 3:**
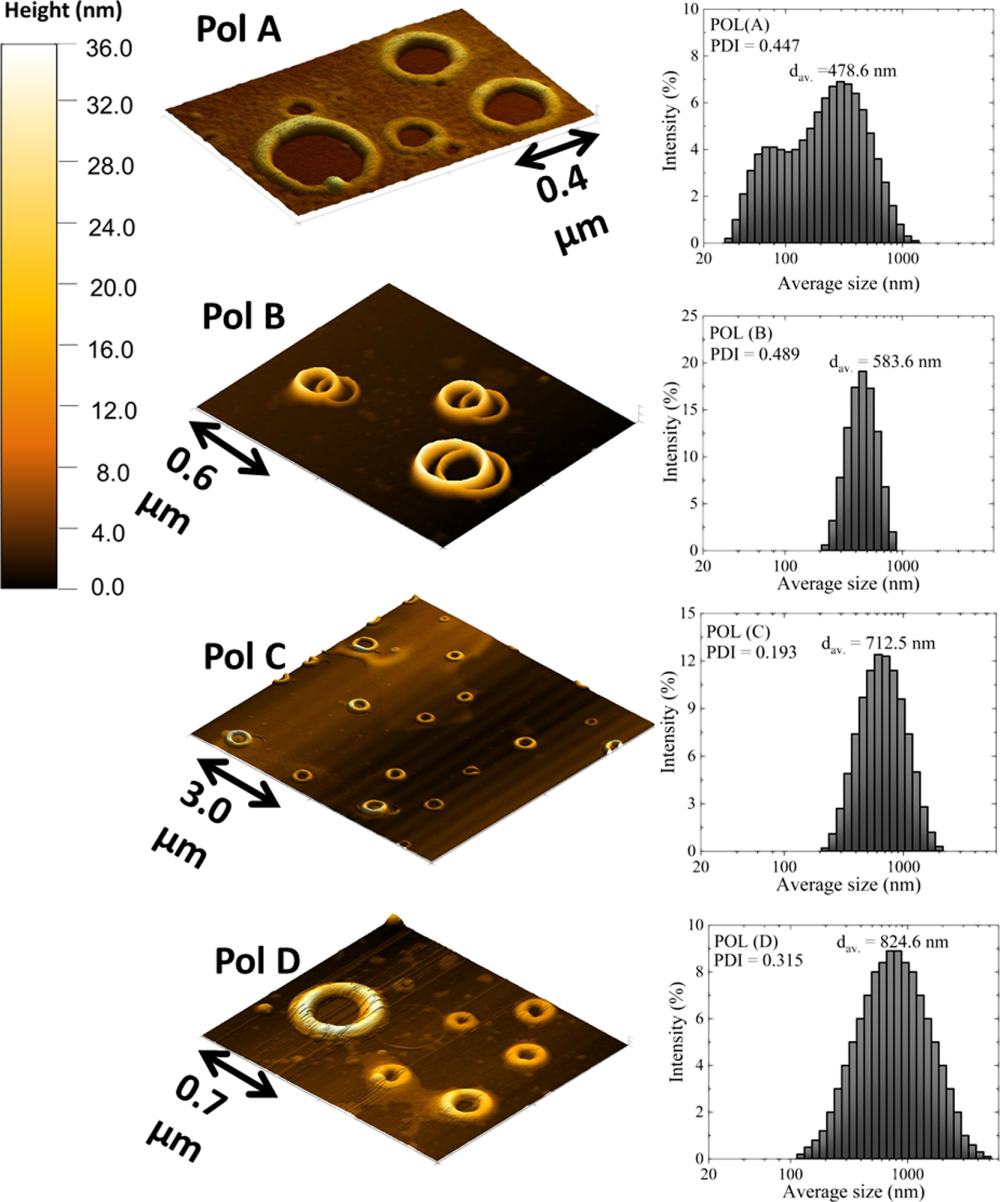
(Left) AFM topography images for CDEP polymers. (Right) Size distribution of the supramolecular structure of the polymers, as obtained from DLS data.

By leveraging the findings of the structural dimensions of the polymers, a more comprehensive analysis was undertaken to understand the correlation between structure, size, and the origin of SHG. The peak values of χ^2^ are plotted as a function of the respective volumes for CDEP structures, and a quadratic tendency is observed in . The nonlinear optical effect generally has a linear dependence on the volume in nanomaterials.^50,51^ This uncommon behavior indicates that it is not just a discrete effect concerning the increase in the structure size. On the other hand, a surface-to-volume analysis shows a minor surface contribution to larger structures, indicating the presence of additional SHG channels beyond symmetry breaking at the interface. As shown in AFM images, all the samples present a structure size in the SHG wavelength dimensions, in which a Quasi-Phase Matching (QPM) process may occur and amplify the nonlinear signal. On the other hand, phase matching happens predominantly in nonlinear optical crystals, where the refraction index for the pump and SHG frequency must be the same (*n*_ω_ = *n*_2ω_) in a broad spectral region, which is not observed in our case. Elshocht et al.^52^ demonstrated that chirality can act as a QPM process, due to the alternation of signals between the enantiomers in Langmuir–Blodgett films of a chiral polymer derived from helicenoquinone. In this case, these results allow us to obtain high χ^(2)^ values without the need to carry out the additional process of inverting second-order nonlinear optical susceptibility values, such as in the polling technique. This periodic inversion of the χ^(2)^ is necessary to compensate for the phase difference of the wave vector (Δ*k⃗* ≠ 0), shifting one phase about the other along the coherence length *l*_c_, which is defined as $l_{c}=\frac{\lambda }{4\left(n_{2\omega }-n_{\omega }\right)}$ ; *l*_c_ generally has dimensions in units of tens of micrometers.^53^

To gain insights into the SHG signal, FDTD simulations^54^ were performed. The structures were modeled as a 3D hollow cylinder inside a continuum medium with a refractive index of 1.48 (DMSO), excited by a Gaussian beam with a width of 8 μm and peak amplitude of 10^10^ V/m, focused on the structure. Given that the beam spot significantly exceeds the dimensions of the structure, the latter will experience an almost uniform electric field. To model the nonlinear optical response, the input χ^(2)^ values were obtained from the results of this work. At the same time, the linear refractive index dispersion was estimated by the Sellmeier model for similar polymers.^55,56^ Then, in the simulated model *n*_ω_ = 1.48 and n_2ω_ = 2.24, the index phase-match condition is not established. Figure 4 illustrates the results obtained for the Pol D sample for a pumping wavelength at λ = 1200 nm. Similar results are found for other polymers. Figure 4a shows the simulation of the magnitude of the 2ω electric field as a function of the position of the sample. The color bar indicates the magnitude of the SHG signal. Figures 4b and 4c were elaborated to understand the additive effect of the magnitude at the 2ω field propagation throughout the structure. In Figure 4b, a longitudinal scan focused on the center of the structure was performed to analyze the 2ω electric field amplitude.

**Figure 4:**
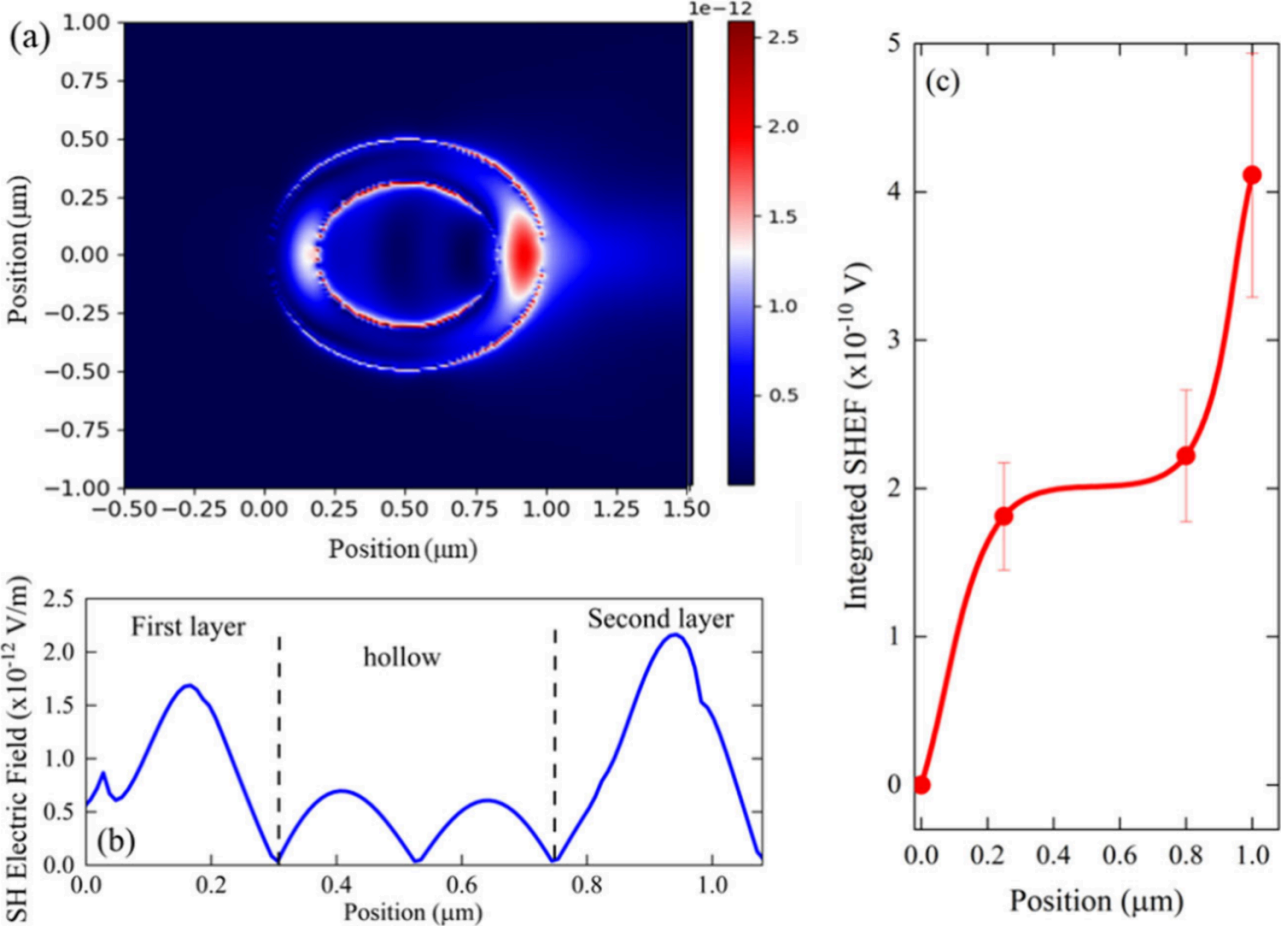
Simulation of the SH optical field generation for the Pol D at a pumping wavelength of 1200 nm. Panel (a) illustrates a color map of the magnitude of the SH electric field as a function of the position of the structure. Panel (b) is a cut in the *y* = 0 plane, showing the oscillation of the field. Panel (c) corresponds to the accumulated magnitude of the SH electric field throughout the structure, divided into sections, where the circles in red are the values of the integral in each section and the continuous line in red shows the trend of the values. The acronym SHEF stands for second harmonic electric field.

As can be seen, a progressive increase of 2ω in the first and second layers indicates a typical QPM process. Also, the 2ω signal is created with high amplitude in the first layer, while, in the hollow part, it decreases, and in the second layer, it is finally amplified. Figure 4c illustrates the integrated signal for each region indicated by the dashed lines in Figure 4b. There is an upward increase in the second harmonic electric field magnitude in the first layer, which remains constant in the hollow part. Therefore, it propagates again through the material, resulting in a considerable increase in the SHG signal via the QFM process. Furthermore, as the wavelength of the 2ω signal generated is approximately an integer of the difference in optical path length, it is suggested that there is the coupling of the field with the structure acting as a nanoresonator,^57–59^ which, in turn, also acts as a mechanism for enhancement of SHG. Notably, no resonances were induced in the structures, likely due to their small diameter, which inhibits them from accommodating high-quality factor resonances for the wavelengths of interest.

Beyond the considerations of structure and shape in a supramolecular medium, chirality also plays a fundamental role in the SHG response. Some studies report that the NLO properties can be enhanced by chirality in supramolecular poled films.^34^ In this medium, the magnetic-dipole interaction significantly contributes to the SHG values compared to the electric dipole one.^60–62^ This phenomenon can be elucidated through the coupling between the backbone and chromophores, which leads to the inclusion of several nonzero tensor components in the second-order optical susceptibility.^63^ To further evaluate the impact of chirality on SHG response, nonlinear optical activity measurements were performed on four samples in DMSO at low concentrations. The SHG technique was used with the insertion of a broadband one-quarter waveplate (λ/4) to control the change in polarization between linear, left (LH), and right-handed (RH) circular polarization before the incidence at the sample. The optical activity dispersion based on the SHG signal (OA-SHG) is illustrated in Figure 5 for all polymers in the DMSO solution. Details about the linear circular dichroism for these polymers can be found in ref 64. In all samples, a slight disparity is observed in the χ^(2)^ values between LH and RH circular polarizations, contingent upon the spectral position of the pump wavelength. One example of it is shown for Pol (A) in the . For 900 nm pump wavelength, displays the statistical measurements for all three polarization states. Besides, the ratio between the dispersion of χ^(2)^ values of circular and linear polarizations $\left(\frac{\chi_{\mathrm{LH}/\mathrm{RH}}^{\left(2\right)}}{\chi_{\mathrm{Linear}}^{\left(2\right)}}\right)$ was $∼\sqrt{2}/2$ . Our results shows the difference between RH and LH circular polarization can ranges from 0.5% to 9%, as can be seen in some examples in , which is undetectable by conventional SHG techniques.

**Figure 5:**
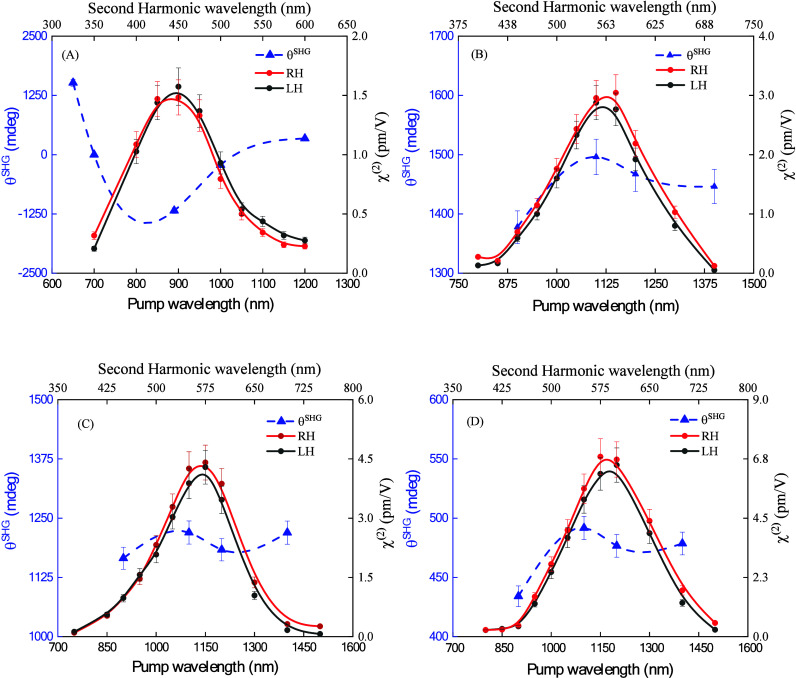
Graphs of OA-SHG dispersion for all samples in DMSO solution. The blue axis on the left represents the θ^SHG^ values, and the blue triangles are the experimental data. The dashed lines are just eye guides. The black axis at the right represents the χ^(2)^ values, where the red and black circles show the experimental data for RH and LH circular polarization. The solid lines are only present to guide the eye.

Therefore, statistical measurements were performed for some pump wavelengths in the range from 625 nm to 1400 nm. The signal of *I*(2ω) was normalized by the intensity of the laser pump (reference signal (ω)) through a lock-in amplifier to avoid variations that do not come from the SHG. Thus, the SHG signal collected by a PMT was integrated for 1 s, and 60 averages were performed. The normalized *I*(2ω) measurements were taken for each sample at the wavelengths studied for each polarization. All the results were normalized by the linear polarization to verify the variation in SHG as a function of the right and left-handed polarizations. Thus, due to the stability of the pumping laser and the experimental system, the standard deviation between the measurements at a given polarization was between 0.5% and 1%.

Chiral nanomaterials may present intriguing features such as negative refractive index superchiral light and thermal realignment, which is interesting in chiral photonics applications.^63,65^ For example, a flexible circularly polarized light (CPL) detector can be developed for potential applications such as security-enhanced, encrypted communications.^66^ Despite this potential interest, gaining knowledge about the difference between RH and LH circular polarization plane angles is valuable. Thus, to quantify the angle of difference between the RH and LH circulars, based on the circular dichroism equation, we employed the following expression:^67^

$$\theta^{\mathrm{SHG}}\;\left(\deg\right)=\left(\frac{180}{\pi }\right){\left(\frac{\sqrt{I{\left(2\omega \right)}_{\mathrm{RH}}^{\mathrm{SHG}}}-\sqrt{I{\left(2\omega \right)}_{\mathrm{LH}}^{\mathrm{SHG}}}}{\sqrt{I{\left(2\omega \right)}_{\mathrm{RH}}^{\mathrm{SHG}}}+\sqrt{I{\left(2\omega \right)}_{\mathrm{LH}}^{\mathrm{SHG}}}}\right)}^{-1}$$

where the terms *I*(2ω)_RH_^SHG^ and *I*(2ω)_LH_^SHG^ are the intensities of the SHG for the RH and LH circular pump polarization, respectively. An example of one of these measurements can be viewed in . For Pol B, Pol C, and Pol D, θ^SHG^ varies around 10% with the following order of magnitude: B > C > D. However, for Pol A, there is a significant variation in the amplitude and maximum at the θ^SHG^ values. The variation in amplitude is approximately 200%, and the difference between the intensities of the circular polarizations can reach around 9%. The inversion in θ^SHG^ values observed between 325 nm and 450 nm is associated with the Cotton effect in the structure of binaphthalene.^68–70^ This effect is related to the combination of circular dichroism and optical activity in the chiral band, which results in a resonance enhancement effect due to the absorption of circularly polarized light. As in Pol A, the second harmonic of the first θ^SHG^ measurement is close to the chiral band; this effect occurs more intensely than in the other polymers. Thus, as the ellipticity value is higher for shorter wavelengths, Pol A has the negative Cotton effect, and the other samples with the opposite wavelength behavior are classified as a positive Cotton effect. Furthermore, with the characterization of this effect, it is possible to connect it to the linear circular dichroism results, in which the positive and negative Cotton effects are linked to the negative and positive linear CD values. Thus, when comparing the results reported in the literature by Koeckelberghs et al.^64^ of the linear CD for the polymers studied here, as well as noting no changes in the spectral positions of the chirality as a function of the chromophores corroborated with the θ^SHG^ results.

Regarding the different θ^SHG^ values between the polymers, in addition to the Cotton effect, a possible explanation may be associated with the proximity of the binaphthalene absorption band to the chromophore absorption band, because chirality is related to the binaphthalene polymer chain. The binaphthalene absorption band is centered at the ultraviolet region (∼350 nm)^43^ and chromophore absorption bands of Pol A, Pol B, Pol C, and Pol D are centered, respectively, 450 nm, 554 nm, 572 and 605 nm, where the Pol A is closer than to other samples, being more susceptible to the influences of chirality at NLO response.

We embarked on a pioneering investigation, delving into the relationship between the structure and SHG dispersion of four novel chiral chromophore-functionalized polybinaphthalenes in a colloidal system. Initially, the dispersion of SHG was unveiled by the SHG technique employing linear polarization. These findings demonstrated a similar dispersion profile and notable disparity in the peak values. The DLS and AFM measurements showed that size and shape play a fundamental role, in addition to the strength of the EWG attached to the central polymer backbone. The rodlike/helical structure determined by AFM associated with the chromophore creates a periodic directional preference throughout the macrostructure for the χ^(2)^ coefficient, suggesting that the SHG origin is associated with a quasi-phase matching process, which is corroborated by FDTD simulations. Moreover, the SHG is enhanced by the 2PA process and by coupling the nonlinear electric field like a nanoresonator. The OA-SHG dispersion measurements distinguish between left-hand (LH) and right-hand (RH) circular polarization. Since chirality is intrinsic to the binaphthalene core, the angle of difference between the RH and LH circular values tends to decrease with a greater redshift in the absorption band of the chromophore. Therefore, such outcomes reveal key insights to lead new strategies for obtaining elevated SHG values at chiral supramolecular derivatives and provide remarkable applications related to the polarized resolved double-frequency conversion process, three-dimensional SHG microscopy at biological systems, and chiral photonics devices.

## Experimental Methods

The supramolecular second-order nonlinear dispersion values were determined by employing the external reference method (ERM), using a femtosecond spectral-resolved SHG technique that employed an optical parametric amplifier (Orpheus - Light Conversion) pumped by an amplified femtosecond laser system (Pharos - Light Conversion, 1030 nm, 190 fs, 7.5 kHz) as tunable laser excitation. The pNA dissolved at DMSO was used as a reference sample. More details can be found in ref 26. The χ^(2)^ values were calculated by normalizing the values of the first-order hyperpolarizability (β) by the volume of each structure. The measurements were performed over a range from 700 nm to 1500 nm with 50 nm intervals for linear and circular polarizations.

The DLS data were measured at concentrations of about 10 μM in pure DMSO in a quartz cuvette with an optical path of 1 cm. The films for AFM measurements were prepared using the spin-coating method on a silicon wafer. More details can be found in .

The TDFD simulations were run for enough time for all the fields to decay and were analyzed in the frequency domain to obtain the field distribution at the pump and SHG frequencies. The simulation volume is surrounded by field monitors, which are used to accumulate and measure the total energy leaving the simulation domain. These field monitors are useful for analyzing SHG energy conversion. Additionally, to effectively truncate the simulations and prevent any unwanted reflections or artifacts, the simulation volume is enclosed by perfectly matched layers (PMLs). These PMLs are designed to absorb the electromagnetic fields exiting the simulation domain, ensuring that the boundaries do not interfere with the simulation results.

## Supplementary Material





## Notes

Absorbance spectra of all samples in dimethyl sulfoxide solution; example of quadratic dependence of the SH signal with the pump laser intensity; example of the linear dependence of the SHG quadratic coefficient with the concentration; description of the 2PA enhancement factor; description of the dynamic light scattering and atomic force microscopy measurements; relationship between χ^(2)^ and volume; example of the SHG signal as a function of the pump laser polarization (PDF)
Transparent Peer Review report available (PDF)
