# Supplementary material for: Strong Second Harmonic Generation and Nonlinear Optical Activity in Chiral Supramolecular Polymers
Source: J Phys Chem Lett. 2025 Dec 11;16(51):13161–9. doi: 10.1021/acs.jpclett.5c02495 (PMC12746451; doi:10.1021/acs.jpclett.5c02495)
Supplement: Supplementary file 1 [file jz5c02495_si_001.pdf]

SUPPORT INFORMATION

Strong Second Harmonic Generation and Nonlinear Optical Activity in Chiral Supramolecular Polymers

Carlos H. D. dos Santos[a], Marcelo.G.Vivas[b], Filipe A. Couto[a], Guy Koeckelberghs[c], Cleber R. Mendonça[a] and Leonardo De Boni[a*]

[a] Instituto de Física de São Carlos, Universidade de São Paulo, 13560-970 São Carlos, SP, Brazil

[b] Laboratório de Espectroscopia Óptica e Fotônica, Universidade Federal de Alfenas, Poços de Caldas, MG, Brazil

[c] Laboratory of Macromolecular and Physical Organic Chemistry, Katholieke Universiteit Leuven, Celestijnenlaan 200F, B-3001 Heverlee, Belgium

* Author to whom correspondence should be addressed: deboni@ifsc.usp.br

1. Linear Absorption

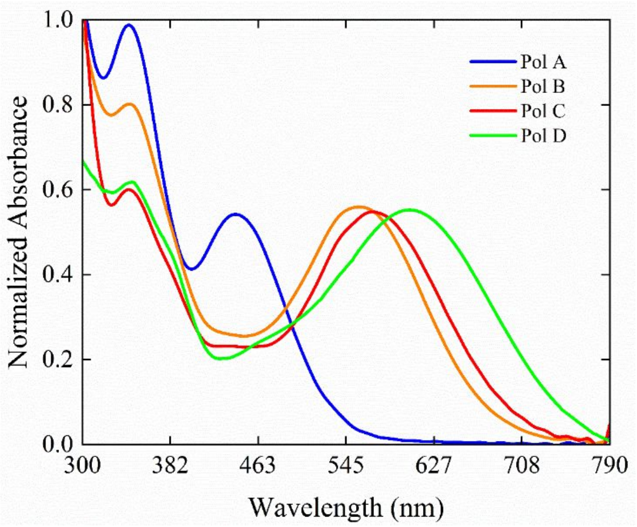

Figure SI-1. Normalized absorbance spectra of the studied samples dissolved in Dimethyl sulfoxide.

2. SHG Curves

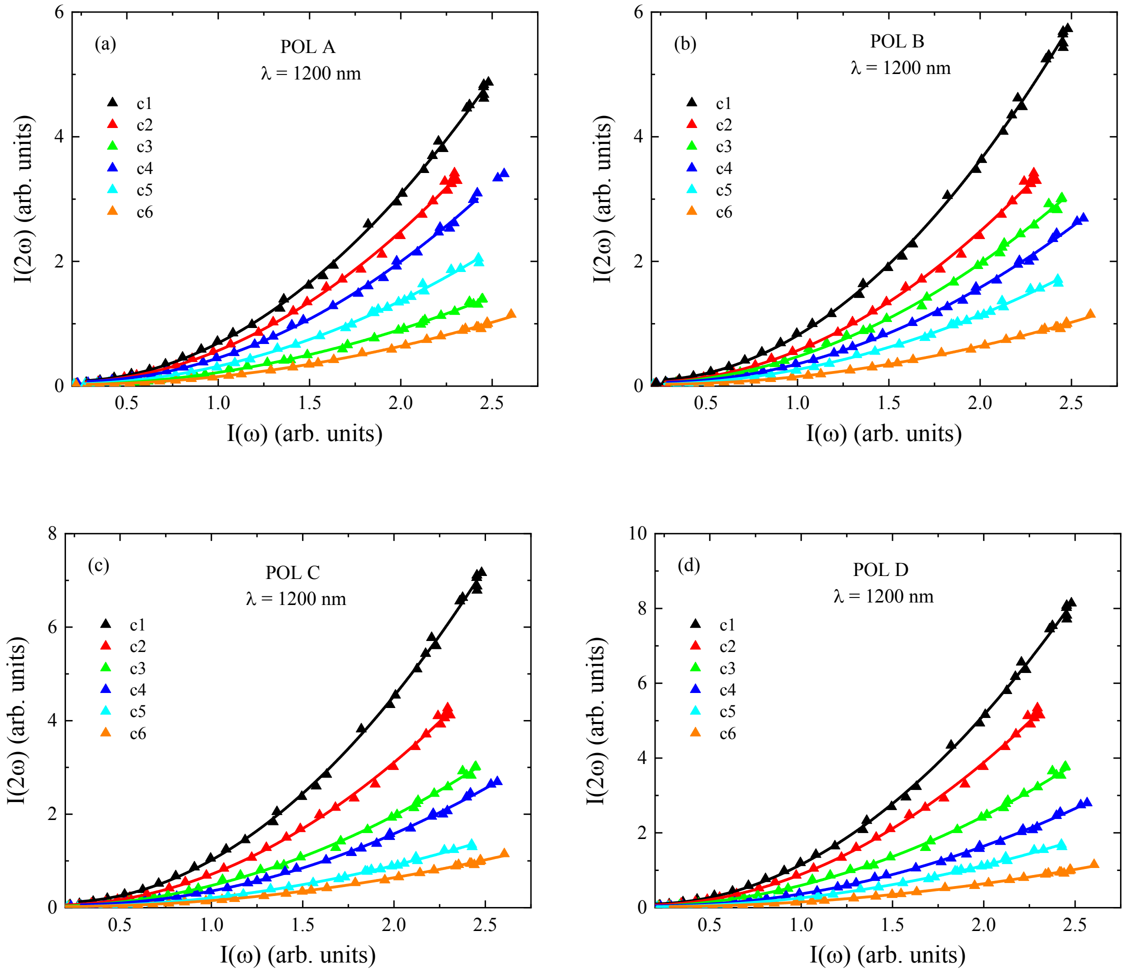

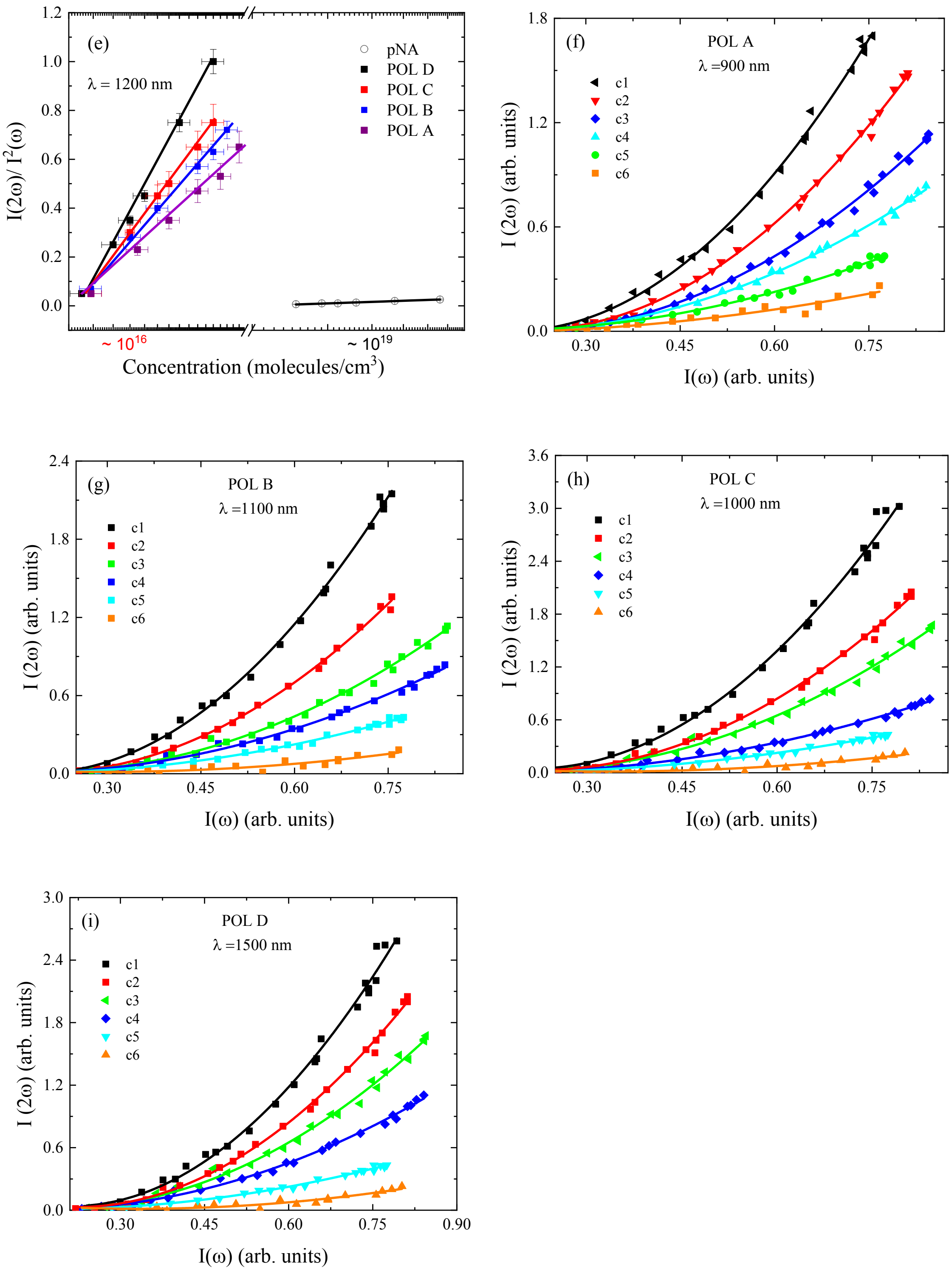

Figure SI-2. (a),(b),(c),(d),(f),(g),(h) and (i) symbols display one example of the measurements of I(2ω) vs I(ω) and their respective quadratic fits for Pol A,B,C and D respectively for different concentrations, measured at different pump wavelengths, in which c1 is the most concentrated solution and the c6 the lowest. (e) depicts the linear dependence of the quadratic coefficient with sample concentration for *para*-Nitroaniline (pNA) and Pol A,B,C and D at 1200 nm, in which the x-axis is in the log scale. This result clearly shows the giant generated 2ω signal from polymers as compared to the pNA organic molecule.

3. 2PA enhancement factor

The 2PA enhancement factor was calculated by the module on following expression:

$$R_{2PA} = \left|\left[\left(\frac{\omega_{01}}{[(\omega_{01} - 2\omega) - i\Gamma_{01}]}\right)\right]\right| \qquad \text{Eq. SI-1}$$

where ω_{01} is the transition frequency related to the chromophore absorption band, Γ_{01} is the respective half-width at half maximum and ω is the laser incident frequency. The photophysics parameters such as ω_{01} and Γ_{01} was obtained by conventional linear absorption spectroscopy.

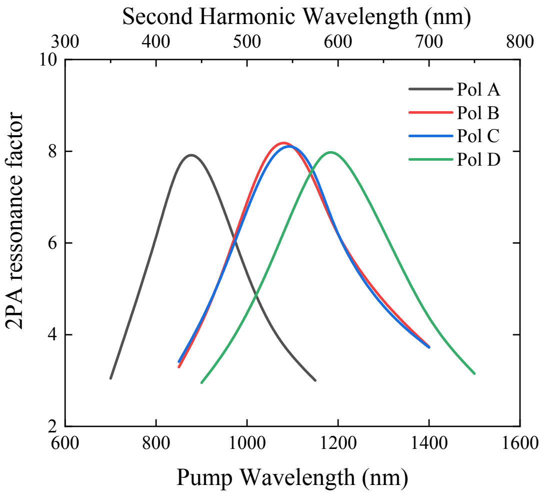

Figure SI-3. Dispersion of the 2PA enhancement factor for the samples studied. Similar peak factors were obtained, differing only in the spectral deviations.

4. Dynamic Light Scattering (DLS)

The DLS measurements were performed on the four polymer samples, each with a 10^{-6} M concentration, dissolved in pure DMSO and subjected to ultrasound for 10 to 20 minutes. These samples were placed into a quartz cuvette with a 1 cm optical path. The equipment used for this task was a *Malvern Panalytical model Zetasizer Pro*, a widely used instrument in the field of polymer science and materials characterization. This instrument utilizes a He-Ne laser with emission centered on 633 nm and a maximum power of 10 mW as a scattering light source. Further details about this equipment can be found on its official website. The particle size detection sensitivity of this system spans from 0.3 to 10 µm, where the scattered light is detected at 90 degrees of incidence by an avalanche photodiode. To ensure accuracy of the results, three batteries were taken for each sample, and an average of 20 measurements was recorded for each battery to calculate the size distribution.

5. Atomic Force Microscopy

Atomic force microscopy (AFM) measurements were also carried out for the four chiral polymers derived from triphenylamine samples. A solution with a 10^{-4} M concentration was prepared using a procedure similar to the DLS experiment. For the deposition of the samples, the spin coating method was used with the *Laurell Technologies model WS-650SZ-6NPP/LITE*. For all the samples, a standard procedure was adopted based on studies carried out in Ref.[2]. A drop of the solution was deposited in the center of the silicon wafer with the spin coater stationary and then accelerated to a speed of 3000 rpm for a total time of approximately 60 seconds. After preparing the sample, it was placed in the AFM equipment for structural analysis. The equipment used was *Nanosurf FLEX-AFM*, which is capable of measuring the topography of a spatial region with nanometric resolutions. In general, measurements start with scans of 10x10 μm regions with a lower resolution (256 points/line). As the regions of interest are found, the area is reduced and the resolution is increased (600 points/line).

6. $\chi^{(2)}$ and volume relation

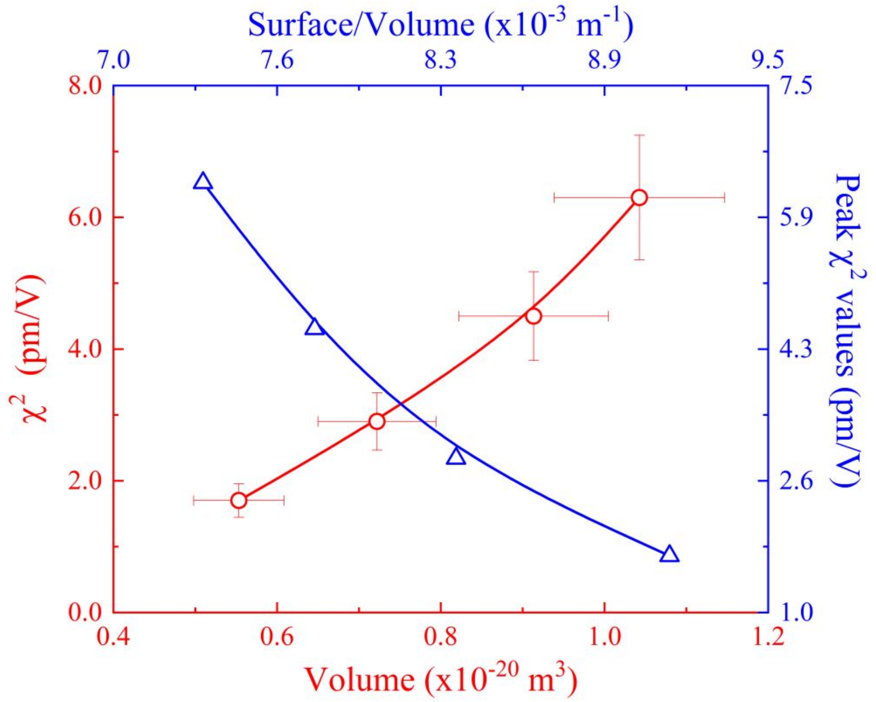

Figure SI-4. The normalized volume χ^2values as a function of volume (bottom red-axis) and surface/volume (top blue-axis). The red circles and blue triangles represent the experimental data, and the solid red line displays a quadratic behavior, indicating other contributions besides increasing volume. The blue solid line is just an eye guide.

7. Chirality SHG measurements

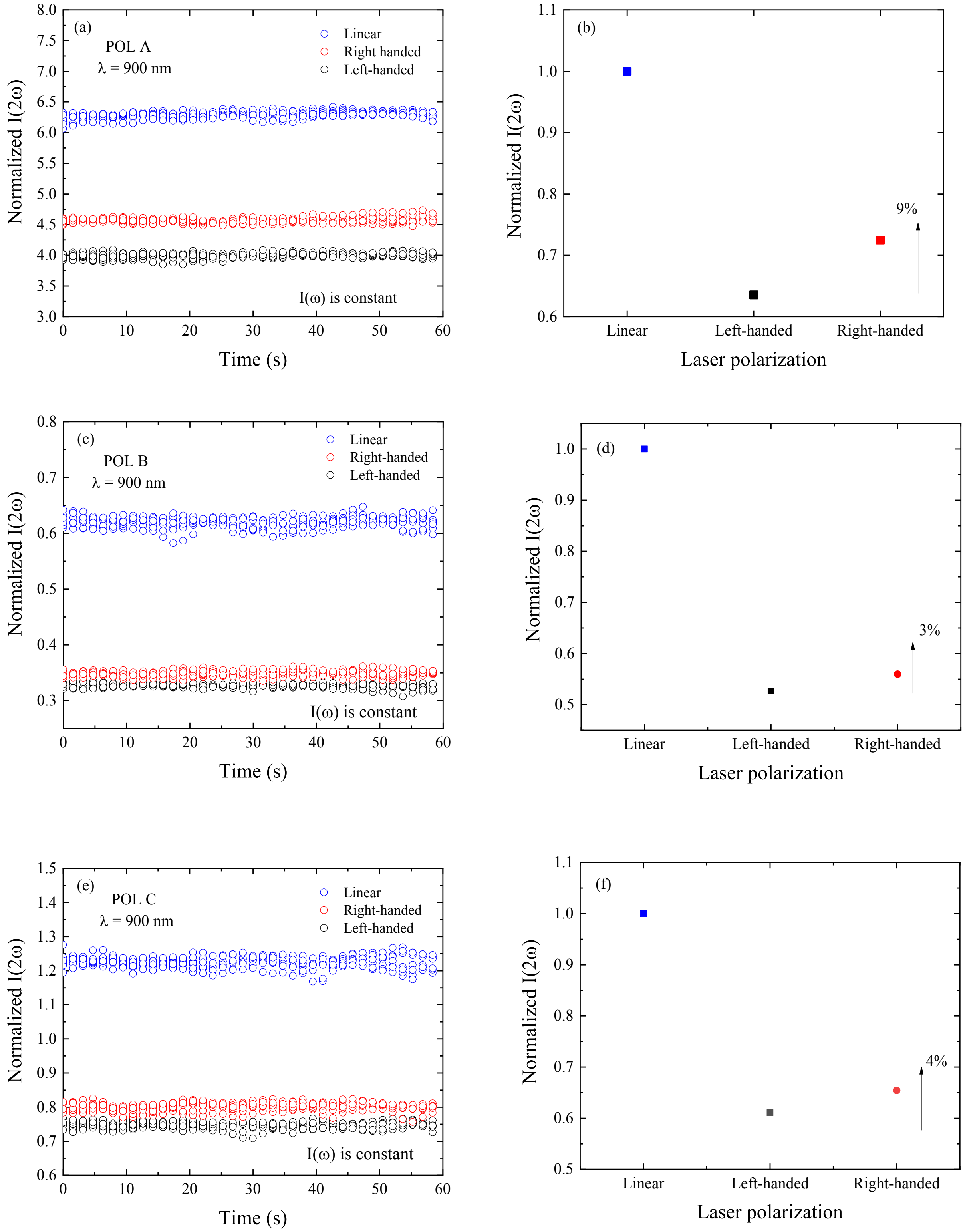

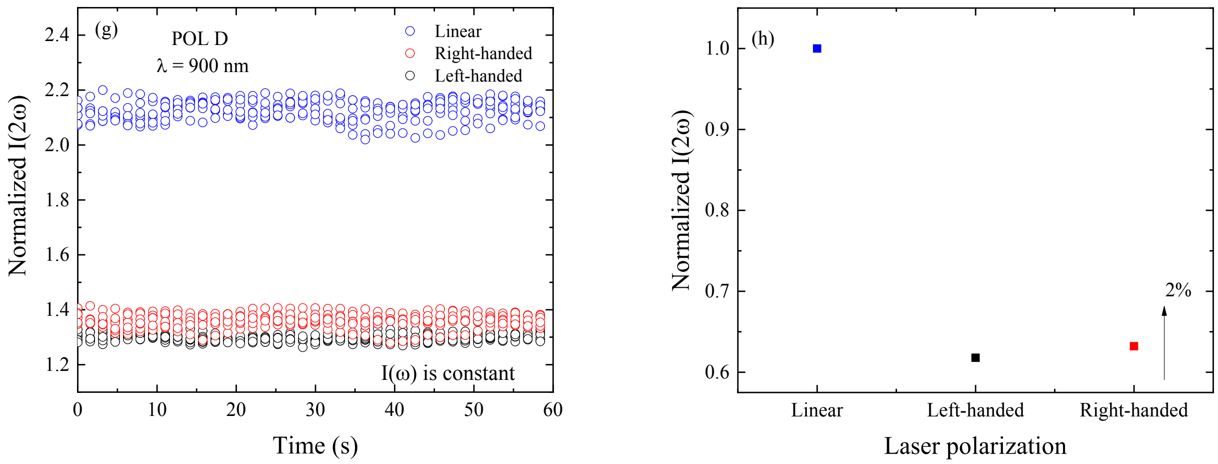

Figure SI-5. Graphs (a),(c),(e) and (g) represents the I(2ω) signal measured at 900 nm normalized for $(I(\omega))^2$ for all three linear, right and left polarizations at the same laser pump intensity. Graphs (b),(d),(f) and (h), the y-axis represents the average values of I(2ω) in each pumping polarization normalized by the linear one.

References

(1) Dipold, J.; Vivas, M. G.; Koeckelberghs, G.; Siqueira, J. P.; De Boni, L.; Mendonca, C. R. Probing the Strong Near-IR Two-Photon Transition in Supramolecular Triphenylamine-Based Polymers by Nonlinear Absorption Spectroscopy. *The Journal of Physical Chemistry B* **2020**, *124* (28), 6147–6153. https://doi.org/10.1021/acs.jpcb.0c04127.

(2) Hall, D. B.; Underhill, P.; Torkelson, J. M. Spin Coating of Thin and Ultrathin Polymer Films. *Polymer Engineering & Science* **1998**, *38* (12), 2039–2045. https://doi.org/10.1002/pen.10373.
