# Supplementary material for: Strong Second Harmonic Generation and Nonlinear Optical Activity in Chiral Supramolecular Polymers
Source: J Phys Chem Lett. 2025 Dec 11;16(51):13161–9. doi: 10.1021/acs.jpclett.5c02495 (PMC12746451; doi:10.1021/acs.jpclett.5c02495)
Supplement: Supplementary file 2 [file jz5c02495_si_002.pdf]

jz-2025-024958.R1

Name: Peer Review Information for "Strong Second Harmonic Generation and Nonlinear Optical Activity in Chiral Supramolecular Polymers"

First Round of Reviewer Comments

Reviewer: 1

Comments to the Author

The authors report on the chiral polymers based on the axial chirality of their binaphthalene-subunits and measure significant second-order susceptibility values and subsequent second-harmonic generation. They also show a dependence on the helicity of light on the SHG intensity.

I find this study to be of potential interest to the readership of JPCL, but in order for the conclusions drawn by the authors to be robust – in particular with respect to the origin and validity of the observe polarization-dependent differences, both on the molecular and supramolecular level – I strongly suggest addressing my open questions below.

1. In the introduction of the polymers in Fig1, the chemical structures lack precision in determining the exact structures. E.g. while it is still predictable that rest A is meant to indicate a para-nitro-phenyl rest to be attached to the polymer motif on the left, the bonding site for the other rests listed is in principle undefined. Please properly redraw the connectivity following normal IUPAC standards.

2. What is the handedness of the polymers shown in each case? The authors do not write out the name of their polymer here or indicate if it is an R or an S polymer, depending on the axial chirality of the binaphthyl and its connectivity.

3. The authors should definitely measure and report the linear circular dichroism of their respective samples (in both ellipticity and gabs) to properly characterize their samples and then build the non-linear responses on. This relates to the polymers in solution and their supramolecular assemblies in the solid, so as to allow for disentangling the helicities on each hierarchical level of chirality.

4. As a sanity check, at least for one of the 4 materials, the authors should also show the other handedness enantiomer and measure its properties in comparison (Cotton effect on the polymer level, but perhaps not on the supramolecular level).

5. In Fig.5, about the OA-SHG of the samples A-D: The data points for RH and LH light overlap within their error bars massively. While the inversion of chiroptical response between A and B/C/D could originate from the supramolecular chirality adding more weight to the signal than the molecular chirality (which should follow the same handedness for all samples due to the identical enantiomer as polymer backbone), I wonder if the analysis presented here is really robust, given the large noise here. Can the authors please comment on this, their S/N and reproducibility of these results? See my point above also, on measuring the CD of the assembly vs. that of the polymer in solution and determine their CD response (and sign, to begin with).

6. In Fig. 5, bottom left panel, there is a typo in the y-axis label (superscript SHG, not GSH); please correct.

7. In the SI, in Fig. SI2: The authors use arbitrary units to quantify the intensity of their SHG-to-fundamental intensity ratio. I find this inappropriate as it can and should be very clearly quantified in terms of a laser fluence.

8. The authors also only showed error bars in the concentration in Fig. SI2 right; Error bars for the measured laser fluences should be added, as they add uncertainties, especially at low fluences.

9. The authors should also add all the raw data for the other materials to the SI, not just for the one example.

10. In Fig. SI3 it looks like those curves were continuous data traces, although they are based on discrete measurement points. Those should be plotted more clearly.

11. In Fig. SI5 left, again no absolute fluence is given, which is crucial to state for reproducibility.

Reviewer: 2

Comments to the Author

This study focuses on potential of chiral supramolecular polymers for advanced optical materials and nonlinear photonics. The authors explore the origin and optical activity of SHG in chiral polybinaphthalenes , using femtosecond laser. Key findings include promising second-order optical from chromophores attached to the polymer backbone. Structural analysis revealed rod-like structures that promote a directional preference for the SHG signal, with simulations indicating a connection to the quasi-phase matching process .

Although this article presents significant findings regarding second harmonic generation in chiral supramolecular polymers, several areas could be discussed in greater detail:

- The article states $\chi(2)\chi(2)$ values are influenced by attached chromophores, A more in-depth discussion on the influence of each acceptor group on SHG would be beneficial, and how their arrangement within the polymer influences the overall nonlinear response, and the interplay between the chromophore's properties and the polymer's helical structure.
- Further discussion on how these chiral supramolecular polymers facilitate QPM would strengthen this point. A comparison with other QPM strategies in different material systems could also provide valuable context.

- The article mentions that 2PA transitions in the near-infrared region promote SHG enhancement. A more detailed explanation of how the moderate 2PA cross-section of these polymers contributes to the observed SHG values, and how the spectral positions of 2PA bands among different polymers lead to resonance enhancement, would be useful. The authors could explore the relationship between the 2PA spectrum and the SHG dispersion curves more explicitly.
- DLS and AFM measurements showed that size and shape play a fundamental role and revealed a hollow cylindrical structure, a more explicit correlation between specific structural parameters and the observed SHG and optical activity values would be useful.
- A discussion on how this polydispersion might affect the uniformity and reproducibility of the optical properties, and potential strategies to reduce it, could be relevant for future applications.
- The conclusion states briefly that these structures are excellent candidates for chiral photonics applications. Providing more concrete examples or potential pathways for their use would enhance the article's perspectives aspect.

Author's Response to Peer Review Comments:

Dear Editor,

We have enclosed the responses to the reviewer's questions on the Questions Reviewer JPCL.pdf file.

Thank you very much for all the help you and your team provided us during the submission and review process.

Best regards,

Leonardo

Reviewer(s)' Comments to Author:

Reviewer: 1

Recommendation: This paper may be publishable, but major revision is needed; I would like to be invited to review any future revision.

Comments: The authors report on the chiral polymers based on the axial chirality of their binaphthalene-subunits and measure significant second-order susceptibility values and subsequent second-harmonic generation. They also show a dependence on the helicity of light on the SHG intensity. I find this study to be of potential interest to the readership of JPCL, but for the conclusions drawn by the authors to be robust – in particular with respect to the origin and validity of the observed polarizationdependent differences, both on the molecular and supramolecular level – I strongly suggest addressing my open questions below.

1. In the introduction of the polymers in Fig1, the chemical structures lack precision in determining the exact structures. E.g. while it is still predictable that rest A is meant to indicate a para-nitro-phenyl rest to be attached to the polymer motif on the left, the bonding site for the other rests listed is in principle undefined. Please properly redraw the connectivity following normal IUPAC standards.

We would like to thank the reviewer for their careful and constructive comments. Figure 1 was recreated based on the article that describes the synthesis of the polymers[1], which was developed by this paper's co-author (Dr. Guy Koeckelberghs), according to IUPAC standards. The modification was done and incorporated into the revised manuscript to more clearly understand the monomer molecular structure for the JPCL reader.

2. What is the handedness of the polymers shown in each case? The authors do not write out the name of their polymer here or indicate if it is an R or an S polymer, depending on the axial chirality of the binaphthyl and its connectivity.

Sorry for the lack of this information. We have added the handedness of studied polymers to the revised manuscript at page 3 highlighted at yellow as follows: “Figure 1 displays the molecular structure of the chiral supramolecular polymers, based on a triphenylamine group as a core (blue) linked to the two-chiral binaphthalene (S enantiomer) units in gray and functionalized chromophores, represented by red color, as can be seen in Figure 1. The details of the synthesis and linear circular dichroism measurements are described in Ref.[2].”

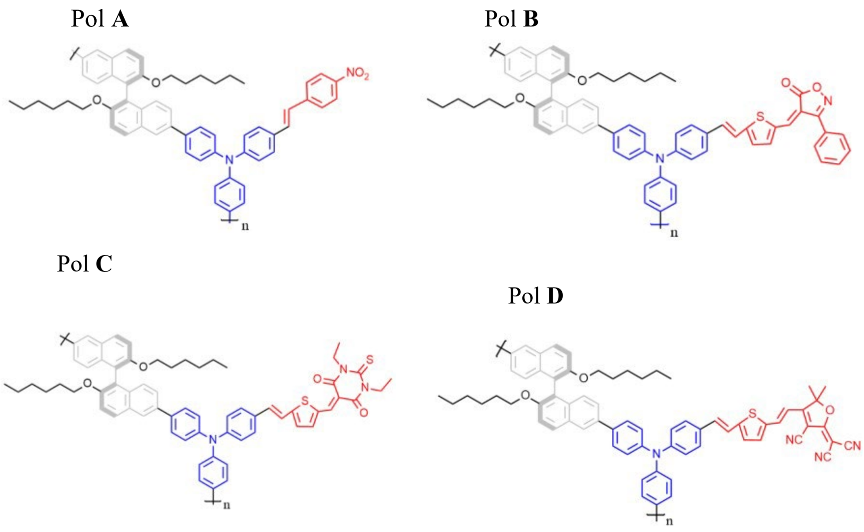

3. The authors should definitely measure and report the linear circular dichroism of their respective samples (in both ellipticity and gabs) to properly characterize their samples and then build the non-linear responses on. This relates to the polymers in solution and their supramolecular assemblies in the solid, so as to allow for disentangling the helicities on each hierarchical level of chirality.

Thank you for the relevant observation. The information about the linear optical chirality of the studied polymers, which are associated with the binaphthalene molecule, is reported in several published papers, and especially in those carried out by Dr. Guy Koeckelberghs[2–4]. The inclusion of studied electron-withdrawing groups (EWG groups) does not influence the polymer's chirality as observed at UV-Vis spectra reported at the SI section. In addition, the results reported for the secondorder nonlinear optical activity indicate the chiral nature of the studied polymers I, in fact, S enantiomers.

4. As a sanity check, at least for one of the 4 materials, the authors should also show the other handedness enantiomer and measure its properties in comparison (Cotton effect on the polymer level, but perhaps not on the supramolecular level).

Thank you for this valuable observation. In this study, we synthesized only the S enantiomer. We did not perform experiments with the other-handedness enantiomer because different enantiomers have the same achiral physical properties. Therefore, the degree of non-centrosymmetry, which governs the SHG signal, is not affected in its amplitude (because nonlinear dichroism is related to the quadrupolar contribution) by the handedness of the enantiomer.

5. In Fig.5, about the OA-SHG of the samples A-D: The data points for RH and LH light overlap within their error bars massively. While the inversion of chiroptical response between A and B/C/D could originate from the supramolecular chirality adding more weight to the signal than the molecular chirality (which should follow the same handedness for all samples due to the identical enantiomer as polymer backbone), I wonder if the analysis presented here is really robust, given the large noise here. Can the authors please comment on this, their S/N and reproducibility of these results? See my point above also, on measuring the CD of the assembly vs. that of the polymer in solution and determine their CD response (and sign, to begin with).

In fact, with the conventional HRS technique, the standard deviation of the measurements, which depends on laser fluctuation, concentration, to cite a few, is about 10%. Therefore, as the reviewer pointed out, there is a large overlap of the SHG signal for right- and left-hand circular polarization within the standard deviation of the measurements. To solve this problem, we implemented the HRS technique with lock-in detection. For each pump wavelength and polarization (linear, rightcircular, and left-circular), we conducted approximately 10 measurements. Each measurement consisted of 60 data points, with each point being integrated by a lockin amplifier that averages around 10,000 pulses per second. It is important to note that the $I_{2\omega}$ is normalized by the I_{ω} to prevent fluctuations from affecting the results. With this, the standard deviation between the measurements at a given polarization was less than 1%, as shown by the

fluctuation data in Figure R1. Figure R1 represents the Pol A measurement at 900 nm pump wavelength with the respective values for linear, left, and right-handed: (6.30 ± 0.03), (4.58 ± 0.03) and (4.01 ± 0.02). Therefore, the standard deviation for OA-SHG measurements is between 0.5 and 1% for all samples, resulting in 20 times more resolution than the HRS conventional technique. So, for Pol A, the error bar is negligible because of the scale of the graph, a consequence of the Cotton effect. However, for the other samples, the measured values are quite close, with a maximum variation of only 10%. They remain nearly constant for SHG wavelengths longer than 500 nm, which aligns with the spectral profile of Pol A. This consistency is also observed in the circular dichroism data from Guy's previous work, as cited in previously asked questions, within the same wavelength range.[1,3–5] Ultimately, the main objective of this study was to demonstrate that the SHG intensity is dependent on polarization and can be detected by performing the proposed lock-in amplification method. This difference is not discernible with the conventional HRS technique.

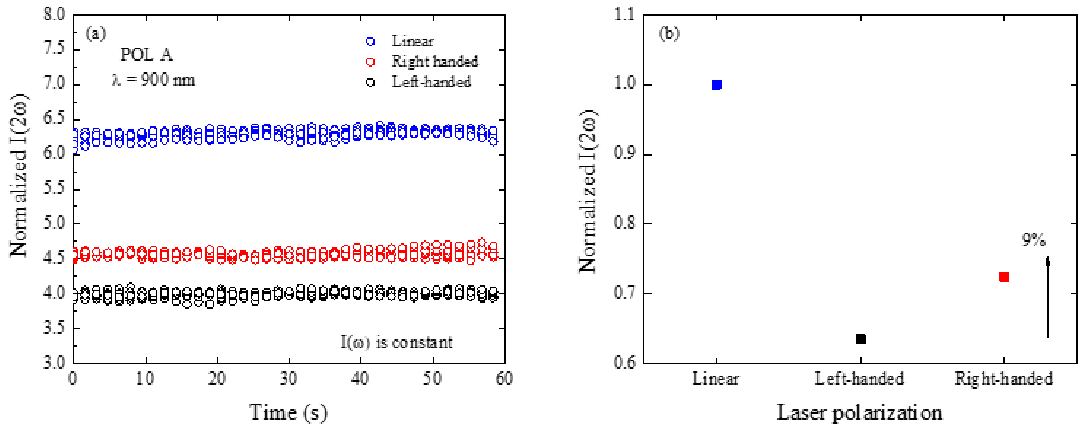

Figure R1. At left, detection via lock-in amplifier for $I_{2\omega}$ for different polarizations for Pol A at 900 nm. At right, normalized $I_{2\omega}$ as a function of incident polarization. Note that with this type of detection, it is possible to identify the difference between polarizations that are not possible via the HRS technique.

6. In Fig. 5, bottom left panel, there is a typo in the y-axis label (superscript SHG, not GSH); please correct.

Thank you for your observation. The graph was corrected.

7. In the SI, in Fig. SI2: The authors use arbitrary units to quantify the intensity of their SHG-to-fundamental intensity ratio. I find this inappropriate as it can and should be very clearly quantified in terms of a laser fluence.

In the literature, it is common practice to represent the I(2ω) curve as a function of I(ω) for experimental data related to SHG and hyper-Rayleigh scattering.

From this data, the parabolic coefficient, which is directly related to the first-order molecular hyperpolarizability coefficient, can be extracted using the external reference method[6–10]. This approach eliminates the need to calculate the laser fluence. The peak intensities used are in the order of tens of GW/cm^2, focusing with a cylinder lens, because the SHG conversion without phase matching is extremely low (in the order of 10^{-13} I_{input}). In this context, it is necessary to use a photomultiplier, as mentioned in our experimental setup.

8. The authors also only showed error bars in the concentration in Fig. SI2 right; Error bars for the measured laser fluences should be added, as they add uncertainties, especially at low fluences.

Thanks for your observation. The graph was corrected.

9. The authors should also add all the raw data for the other materials to the SI, not just for the one example.

In this study, the hyperpolarizability dispersion was measured for four samples, each with an average of 40 experimental parabolic curves (10 for each polarization plus 10 from the reference sample, totaling 160 curves). Therefore, we believe there may not be a need to show all the curves, as they represent the same profile. However, we have added a few more curves to Figure SI-2, one from each sample.

10. In Fig. SI3 it looks like those curves were continuous data traces, although they are based on discrete measurement points. Those should be plotted more clearly.

Figure SI.3 is a numerical simulation performing the two-level model[9,11] to demonstrate that the two-photon absorption enhancement is similar across all studied samples. As these are simulation data, we believe the best way to present them is with a continuous line.

11. In Fig. SI5 left, again no absolute fluence is given, which is crucial to state for reproducibility.

Thanks for your observation. This point was clarified in the answer to question 7.

#Reviewer: 2

Recommendation: This paper may be publishable, but major revision is needed; I would like to be invited to review any future revision.

Comments: This study focuses on the potential of chiral supramolecular polymers for advanced optical materials and nonlinear photonics. The authors explore the origin and optical activity of SHG in chiral polybinaphthalenes , using a femtosecond laser. Key findings include promising second-order optical properties from chromophores attached to the polymer backbone. Structural analysis revealed rod-like structures that promote a directional preference for the SHG signal, with simulations indicating a connection to the quasi-phase matching process. Although this article presents significant findings regarding second harmonic generation in chiral supramolecular polymers, several areas could be discussed in greater detail:

1) The article states χ(2) values are influenced by attached chromophores, A more in-depth discussion on the influence of each acceptor group on SHG would be beneficial, and how their arrangement within the polymer influences the overall nonlinear response, and the interplay between the chromophore's properties and the polymer's helical structure.

We would like to thank the reviewer for their careful and constructive comments. A new paragraph has been added on page 3, highlighted in yellow.

"It is worth mentioning that $\chi^{(2)} \propto N\mu\beta E$, in which N is the concentration of chromophore, μ is the dipole moment, β is the first-order hyperpolarizability coefficient, and E is the applied electric field. In this case, it implies that the $\chi^{(2)}$ signal can be increased using chromophores with higher hyperpolarizabilities and dipole moments or by increasing the chromophore concentration.

2) Further discussion on how these chiral supramolecular polymers facilitate QPM would strengthen this point. A comparison with other QPM strategies in different material systems could also provide valuable context.

A new paragraph has been added on page 3, highlighted in yellow.

"Besides, the attachment of the chromophores as a side chain results in a treelike structure that prevents the undesired centrosymmetrical ordering of the chromophores, but becomes flexible enough to induce the noncentrosymmetry by electrical poling[3]."

3) The article mentions that 2PA transitions in the near-infrared region promote SHG enhancement. A more detailed explanation of how the moderate 2PA cross-section of these polymers contributes to the observed SHG values, and how the spectral positions of 2PA bands among different polymers lead to resonance enhancement, would be useful. The authors could explore the relationship between the 2PA spectrum and the SHG dispersion curves more explicitly.

In fact, when comparing SHG results from different chromophores, it is important to consider the relative position of the one- and two-photon absorption bands of the materials because the difference between the observed signals may be due only to effects of the spectral position of the electronic transitions of the materials and not to their hyperpolarizability[12,13] . In this context, a new paragraph has been added on page 5, highlighted in yellow, to clarify this point.

"Initially, the high χ^2 peak values could be attributed to the two-photon absorption (2PA) transition in the near-infrared region,[3] which promotes enhancement in the SHG

signal due to the two-photon resonance, as described by the second-order time-dependent perturbation theory.[9] However, these polymers present a moderate 2PA cross-section compared to other π-conjugated polymers. At the same time, the variance in the SHG maxima, reaching up to three-fold when comparing Pol A with Pol D, cannot be explained only by considering the 2PA. Another effect would be the different spectral positions of 2PA bands among the polymers, which could favor a resonance enhancement effect on the SHG signal. To understand this, a two-level model (2LM)[11] simulation for the LE absorption band (chromophore absorption band) was employed to calculate the 2PA enhancement factor that relates to the enhancement of SHG when the frequency of the scattered photon (2ω) is close to the 2PA allowed transition[14]. Using the second-order perturbation theory, we can see clearly this effect because the denominator in the dynamical term for 2LM is proportional to $(\omega_{01} - 2\omega)$, in which ω_{01} is the two-photon allowed transition frequency of chromophore (see SI).

4) DLS and AFM measurements showed that size and shape play a fundamental role and revealed a hollow cylindrical structure, a more explicit correlation between specific structural parameters and the observed SHG and optical activity values would be useful.

Thank you for your observation. Some points regarding size and shape related to SHG have been addressed in the manuscript. The AFM images reveal the size and shape of polymers studied and confirmed in solution by DLS measurements. In Figure R2 (see Figure SI-4 at Support Information), we plotted the peak $\chi^{(2)}$ values as a function of the structure's volume. The results reveal that the SHG signal has a quadratic dependence on the supramolecular structure volume. On the other hand, for example, linear absorption of nanomaterials has only a linear dependence. These results indicate that the supramolecular structure volume has a strong dependence on the SHG signal. This uncommon behavior indicates that it is not just a discrete effect concerning the increase of the structure size. Finally, it was possible to suggest and perform a simulation of QPM process as explained in the manuscript.

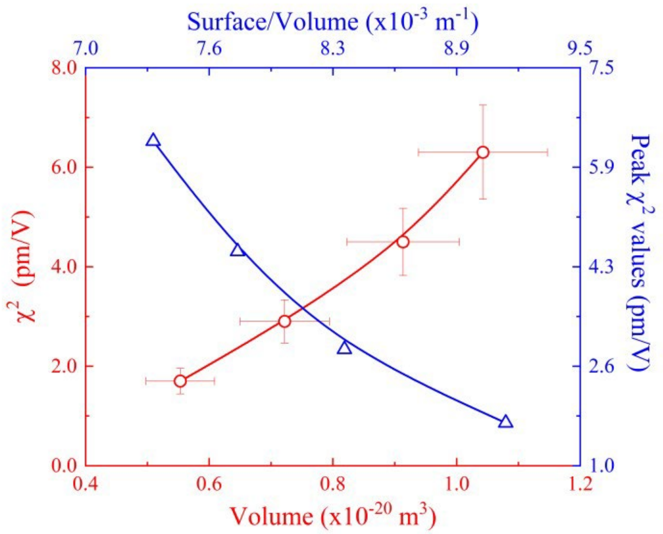

Figure R2. The normalized volume χ^2 values as a function of volume (bottom redaxis) and surface/volume (top blue-axis). The red circles and blue triangles represent the experimental data, and the solid red line displays quadratic behavior, indicating other contributions besides increasing volume. The blue solid lines are just eye guides.

5) A discussion on how this polydispersion might affect the uniformity and reproducibility of the optical properties, and potential strategies to reduce it, could be relevant for future applications.

A new paragraph has been added on page 5, highlighted in yellow, to clarify this point.

"As can be seen in DLS measurements, the average size of the size distribution presents a progressive increase, going from 478 nm (Pol A) to 825 nm (Pol D), with an average polydispersity index (PDI) value around 0.3, indicating a considerable level of polydispersity. In general, if PDI > 0.5, the distribution is rather broad and could significantly increase in the linear scattering in solution that affects the SHG conversion and consequently the optical activity signal in studied samples".

6) The conclusion states briefly that these structures are excellent candidates for chiral photonics applications. Providing more concrete examples or potential pathways for their use would enhance the article's perspectives aspect.

A new paragraph and citations have been added on page 10, highlighted in yellow, to clarify this point.

"Chiral nanomaterials may present intriguing features such as negative refractive index, superchiral light, and thermal realignment, which is interesting in chiral photonics applications.[15,16] As an example, a flexible circularly polarized light (CPL) detectors can be developed for potential applications such as security-enhanced, encrypted communications[17]. Despite this potential interest, gaining knowledge about the difference between RH and LH circular polarization plane angles is valuable".

References

[1] G. Koeckelberghs, S. Sioncke, T. Verbiest, A. Persoons, and C. Samyn, "Synthesis and Properties of Chiral Donor-Embedded Polybinaphthalenes for Nonlinear Optical Applications," Chemistry of Materials **15**(15), 2870–2872 (2003).

[2] G. Koeckelberghs, M. Vangheluwe, I. Picard, L. De Groof, T. Verbiest, A. Persoons, and C. Samyn, "Synthesis and Properties of New Chiral Donor-Embedded

Polybinaphthalenes for Nonlinear Optical Applications," Macromolecules **37**(23), 8530–8537 (2004).

[3] G. Koeckelberghs, S. Sioncke, T. Verbiest, I. Van Severen, I. Picard, A. Persoons, and C. Samyn, "Synthesis and Properties of Chiral Chromophore-Functionalized Polybinaphthalenes for Nonlinear Optics: Influence of Chromophore Concentration," Macromolecules **36**(26), 9736–9741 (2003).

[4] G. Koeckelberghs, S. Sioncke, T. Verbiest, A. Persoons, and C. Samyn, "Synthesis and properties of chiral helical chromophore-functionalised polybinaphthalenes for secondorder nonlinear optical applications," Polymer (Guildf) **44**(14), 3785–3794 (2003).

[5] G. Koeckelberghs, T. Verbiest, M. Vangheluwe, L. De Groof, I. Asselberghs, I. Picard, K. Clays, A. Persoons, and C. Samyn, "Influence of Monomer Optical Purity on the Conformation and Properties of Chiral, Donor-Embedded Polybinaphthalenes for Nonlinear Optical Purposes," Chemistry of Materials **17**(1), 118–121 (2005).

[6] K. Clays, and A. Persoons, "Hyper-Rayleigh scattering in solution," Phys Rev Lett **66**(23), 2980–2983 (1991).

[7] K.J. Clays, E. Hendrickx, S. Houbrechts, M. Triest, T. Verbiest, A.P. Persoons, and C. Samyn, "Characterization of polymeric nonlinear optical materials by hyper-Rayleigh scattering in solution," edited by G.R. Moehlmann, (1993), p. 182.

[8] T. Verbiest, K. Clays, and V. Rodriguez, *Second-Order Nonlinear Optical Characterization Techniques* (CRC Press, 2009).

[9] C.H.D. dos Santos, L.H. Zucolotto Cocca, A.G. Pelosi, V.F. Batista, D.C.G.A. Pinto, M.A.F. Faustino, M.G. Vivas, J. de Paula Siqueira, C.R. Mendonça, and L. De Boni, "Observation of the two-photon transition enhanced first hyperpolarizability spectra in cinnamaldehyde derivatives: A femtosecond regime study," J Chem Phys **158**(21), (2023).

[10] C.R. J.L.CLABEL; DOS SANTOS, CARLOS H.D.; DE BONI, LEONARDO;

MENDONÇA, "Second Harmonic Genertion in perovskite particles for bioimaging: unveiling the effects of particle size and structural modification," (2024).

[11] L.F. Sciuti, L.M.G. Abegão, C.H.D. dos Santos, L.H. Zucolotto Cocca, R.G.M. da Costa, J. Limberger, L. Misoguti, C.R. Mendonça, and L. De Boni, "Modeling the First-Order Molecular Hyperpolarizability Dispersion from Experimentally Obtained One- and TwoPhoton Absorption," J Phys Chem A **126**(14), 2152–2159 (2022).

[12] D.L. Silva, R.D. Fonseca, M.G. Vivas, E. Ishow, S. Canuto, C.R. Mendonca, and L. De Boni, "Experimental and theoretical investigation of the first-order hyperpolarizability of a class of triarylamine derivatives," J Chem Phys **142**(6), (2015).

[13] M.G. Vivas, D.L. Silva, R.D.F. Rodriguez, S. Canuto, J. Malinge, E. Ishow, C.R. Mendonca, and L. De Boni, "Interpreting the First-Order Electronic Hyperpolarizability for a Series of Octupolar Push–Pull Triarylamine Molecules Containing Trifluoromethyl," The Journal of Physical Chemistry C **119**(22), 12589–12597 (2015).

[14] C.H.D. dos Santos, L.H. Zucolotto Cocca, A.G. Pelosi, V.F. Batista, D.C.G.A. Pinto, M.A.F. Faustino, M.G. Vivas, J. de Paula Siqueira, C.R. Mendonça, and L. De Boni, "Observation of the two-photon transition enhanced first hyperpolarizability spectra in cinnamaldehyde derivatives: A femtosecond regime study," J Chem Phys **158**(21), (2023).

[15] S. Sioncke, T. Verbiest, and A. Persoons, "Second-order nonlinear optical properties of chiral materials," Materials Science and Engineering: R: Reports **42**(5–6), 115–155 (2003).

[16] B. Nowacki, H. Oh, C. Zanlorenzi, H. Jee, A. Baev, P.N. Prasad, and L. Akcelrud, "Design and Synthesis of Polymers for Chiral Photonics," Macromolecules **46**(18), 7158– 7165 (2013).

[17] K. Gao, S. Kim, W. Zhao, X. Ye, P. Wang, L. Liu, J. Ahn, H. Zhuo, Z. Li, Z. Wang, G. Chang, W. Ma, M. Zhang, G. Long, X. Shang, and J.H. Oh, "High-performance flexible circularly polarized light photodetectors based on chiral n-type naphthalenediimidebithiophene polymers," Npj Flexible Electronics **9**(1), 83 (2025).

jz-2025-024958.R2

Name: Peer Review Information for "Strong Second Harmonic Generation and Nonlinear Optical Activity in Chiral Supramolecular Polymers"

Second Round of Reviewer Comments

Reviewer: 1

Comments to the Author

I am satisfied with the revisions made and can now recommend publication.

Reviewer: 2

Comments to the Author

I accept the corrections

Author's Response to Peer Review Comments:

Dear Editor.

We modified both the manuscript and the support information files, removing any marks.

Thank you very much for the assistance.

Best regards,

Leonardo
